# Betulinic Acid Hydroxamate is Neuroprotective and Induces Protein Phosphatase 2A-Dependent HIF-1α Stabilization and Post-transcriptional Dephosphorylation of Prolyl Hydrolase 2

**DOI:** 10.1007/s13311-021-01089-4

**Published:** 2021-08-02

**Authors:** María E. Prados, Alejandro Correa-Sáez, Juan D. Unciti-Broceta, Martín Garrido-Rodríguez, Carla Jimenez-Jimenez, Massimiliano Mazzone, Alberto Minassi, Giovanni Appendino, Marco A. Calzado, Eduardo Muñoz

**Affiliations:** 1 Emerald Health Biotechnology, Cordoba, Spain; 2 Instituto Maimónides de Investigación Biomédica de Córdoba (IMIBIC), Cordoba, Spain; 3 Department of Cellular Biology, Physiology and Immunology, University of Cordoba, Cordoba, Spain; 4 Hospital Universitario Hospital Reina Sofia, Cordoba, Spain; 5 Laboratory of Tumor Inflammation and Angiogenesis, Center for Cancer Biology, VIB-KULeuven, 3000 Leuven, Belgium; 6 Department of Drug Science, University of Piemonte Orientale, Novara, Italy

**Keywords:** Protein phosphatase A2, Hypoxia-inducing factor, Prolyl-hydroxylases, Neuroprotection, Betulinic acid

## Abstract

Huntington’s disease (HD) is a neurodegenerative disorder characterized by unwanted choreatic movements, behavioral and psychiatric disturbances, and dementia. The activation of the hypoxic response pathway through the pharmacological inhibition of hypoxia-inducing factor (HIF) prolyl-hydroxylases (PHDs) is a promising approach for neurodegenerative diseases, including HD. Herein, we have studied the mechanism of action of the compound Betulinic acid hydroxamate (BAH), a hypoximimetic derivative of betulinic acid, and its efficacy against striatal neurodegeneration using complementary approaches. Firstly, we showed the molecular mechanisms through which BAH modifies the activity of the PHD2 prolyl hydroxylase, thus directly affecting HIF-1α stability. BAH treatment reduces PHD2 phosphorylation on Ser-125 residue, responsible for the control of its hydrolase activity. HIF activation by BAH is inhibited by okadaic acid and LB-100 indicating that a protein phosphatase 2A (PP2A) is implicated in the mechanism of action of BAH. Furthermore, in striatal cells bearing a mutated form of the huntingtin protein, BAH stabilized HIF-1α protein, induced *Vegf* and *Bnip3* gene expression and protected against mitochondrial toxin-induced cytotoxicity. Pharmacokinetic analyses showed that BAH has a good brain penetrability and experiments performed in a mouse model of striatal neurodegeneration induced by 3-nitropropionic acid showed that BAH improved the clinical symptoms. In addition, BAH also prevented neuronal loss, decreased reactive astrogliosis and microglial activation, inhibited the upregulation of proinflammatory markers, and improved antioxidant defenses in the brain. Taken together, our results show BAH’s ability to activate the PP2A/PHD2/HIF pathway, which may have important implications in the treatment of HD and perhaps other neurodegenerative diseases.

**Supplementary Information:**

The online version contains supplementary material available at 10.1007/s13311-021-01089-4.

## Introduction

Terpenoids and especially triterpenoids are the largest and the most diverse group of naturally occurring compounds. Some triterpenoids and their semi-synthetic derivatives can be identified as potential therapeutics against a wide number of diseases [1]. Betulinic acid (BA) is a lupane-type triterpenoid having a special place among naturally derived remedies. Since botanical preparations containing BA as an active substance, it has been used in folk medicine for centuries [2]. Several preclinical studies have shown that BA is endowed with potent neuroprotective activities by targeting different pathways [3–8]. In addition, BA has been widely used to develop novel semi-synthetic derivatives with improved pharmacological attributes [2, 9]. We have recently described that some triterpenoid hydroxamate derivatives including betulinic acid hydroxamate (BAH) activate the hypoxia inducing factor (HIF) pathway and therefore represent promising small molecules for the development of new drug candidates against inflammatory diseases [10, 11].

HIF-1α and HIF-2α are transcription factors stabilized by a cellular low oxygen status (hypoxia). They regulate the expression of a host of genes whose products are involved in biological processes as diverse as erythropoiesis, angiogenesis, vascular tone, and immunity. Molecular oxygen controls the cellular stability of HIF-1α and HIF-2α via HIF prolyl hydroxylases (PHDs), a class of iron-containing dioxygenases that, in the presence of molecular oxygen and 2-oxoglutarate, hydroxylate HIF-1α and HIF-2α, inducing their ubiquitination by an E3-ubiquitin ligase and degradation by the 26S proteasome. Several lines of evidence suggest that the HIF-1α/HIF-2α stabilization induced by pharmacological inhibition of PHDs may be of clinical relevance for the treatment of ischemic and inflammatory conditions [12]. In addition, post-transcriptional modifications of PHD2 by dephosphorylation of Ser-125 have been described to inhibit its function and to stabilize HIF-1α protein [13]. Interestingly, this dephosphorylation seems to be mediated by B55α (PPP2R2A), a subunit of the protein phosphatase 2A (PP2A) complex [13]. Moreover, it has been described recently that B55α/PP2A plays a major role in vascular remodeling [14], which is of special relevance for neuroinflammatory diseases where the blood brain barrier is compromised.

Huntington’s disease (HD) is a fatal autosomal dominant and progressive neurodegenerative disease caused by the expansion of a trinucleotide CAG repeat (> 36 glutamines) located 17 codons downstream of the initiation codon of the Huntingtin gene (Htt) [15]. Mutated Htt protein leads to death and dysfunction of the GABAergic medium spiny striatal neurons, leading to severe neurological symptoms that included chorea, cognitive impairment, and changes in mood and personality [16].

Hypoxia preconditioning induced by mild oxygen depletion or by pharmacological inhibition of PHDs is expected to beneficially affect various neurological disorders including HD [17–20]. In this sense, HIF-1α protects against oxidative stress by directly targeting mitochondria [21]. Indeed, mitochondrial energy metabolism is considered the primary defect in HD. Mitochondrial deficit tilts energy economy towards aerobic glycolysis to compensate the impaired oxidative phosphorylation, and the upregulation of glycolytic enzymes by HIF-1α is a beneficial adaptive modification to cope with an overall impaired aerobic metabolism [16, 22].

An effort to discover small molecules inhibiting PHDs has led to big pharma and academic groups to develop PHDs inhibitors [23], with HD being a challenging additional pharmaceutical target for this class of compounds [15]. Herein we investigated the mechanism of action of betulinic acid hydroxamate (BAH) to inhibit PHD2 activity and demonstrated its efficacy in *in vitro* and *in vivo* models of HD.

## Material and Methods

### Cell Lines and Reagents

HEK-293T and NIH 3T3 cells were maintained at 37 °C in a humidified atmosphere containing 5% CO2 in DMEM supplemented with 10% fetal bovine serum (FBS), 2 mM L-glutamine, and 1% (v/v) penicillin/streptomycin. STHdh^Q7/Q7^ and STHdh^Q111/Q111^ cell lines, which express either a wild type or a mutated form of the huntingtin protein, were cultured at 33 °C and 5% CO_2_ in DMEM supplemented with 10% FBS, 2 mM L-glutamine, and 1% (v/v) penicillin/streptomycin [24]. HA-PHD1 (#18961), HA-PHD2 (#18963), and HA-PHD3 (#18960) plasmids were obtained from Addgene. GST-PHD plasmids were provided by Edurne Berra (CICbioGUNE, Bilbao, Spain). All other reagents were purchased from Merk (St Louis, MO, USA). Scramble control oligonucleotide siRNA non-targeting pool (#D-001810) and ON-TARGET plus SMARTpool against B55α (#L-004824) were purchased from Dharmacon (Waltham, MA, USA).

### Western Blotting and Antibodies

After treatments, the cells were washed with PBS and proteins were extracted in 50 μl of lysis buffer (50 mM Tris–HCl pH 7.5, 150 mM NaCl, 10% glycerol, and 1% NP-40) supplemented with 10 mM NaF, 1 mM Na_3_VO_4_, 10 μg/ml leupeptine, 1 μg/ml pepstatin and aprotinin, and 1 μl/ml PMSF saturated. Seventy micrograms of protein were boiled at 95 °C in Laemmli buffer and electrophoresed in 10% SDS/PAGE gels. Separated proteins were transferred to PVDF membranes (20 V for 30 min) and blocked in TBS solution containing 0.1% Tween 20 and 5% non-fat dry milk for 1 h at room temperature. Immunodetection of specific proteins was carried out by incubation with primary antibody against human HIF-1α (1:1000 dilution, #610,959, BD Biosciences, San Jose, CA, USA), murine HIF-1α (1:1000 dilution, #ab179483, Abcam, Cambridge, UK), PHD1 (1:1000 dilution, #ab108980, Abcam), PHD2 (1:1000 dilution, #ab109088, Abcam), PHD3 (1:1000 dilution, #ab30782, Abcam), OH-HIF-1α (1:1000 dilution, #3434S, Cell Signaling, Danvers, MA, USA), B55α (1:1000 dilution, #5689S, Cell Signaling), anti-HA (1:1000 dilution, clone 3F10 Roche), anti-Phospho-PHD2 Ser-125 (1:500) [13], and β-actin (1:10.000 dilution, #A5316, Merk, St Louis, MO, USA) overnight at 4 °C. After washing membranes, horseradish peroxidase-conjugated secondary antibody was added and detected by chemiluminescence system (GE Healthcare Europe GmbH).

### Cell Transfections and Immunoprecipitations

Transient transfections were performed with Roti-Fect (#P001.4, Carl Roth, Karlsruhe, Germany) and maintained between 36 and 48 h after transfection. DNA amounts in each transfection were kept constant after the addition of an empty expression vector. B55α silencing was performed with Lipofectamine RNAiMax transfection reagent (#13778100, Life Technologies, Carlsbad, USA) according to the manufacturer’s instructions. Cells were collected, washed in PBS, and lysed in IP buffer [25]. After preclearing the cell lysates with protein A/G Sepharose (Santa Cruz), immunoprecipitation was completed on a rotating wheel upon the addition of 1 μg of the indicated antibodies and 25 μl of protein A/G Sepharose beads. Immunoprecipitated proteins were then five times washed in IP buffer and eluted in 2× SDS sample buffer, followed by western blotting.

### Sample Preparation for LC–MS/MS

Beads used in immunoprecipitation were cleaned three times with 500 µl of 200 mM ammonium bicarbonate, and 60 µl of 6 M urea/200 mM ammonium bicarbonate were added. Samples were then reduced with dithiothreitol (30 nmol, 37 °C, 60 min), alkylated in the dark with iodoacetamide (60 nmol, 25 °C, 30 min), and diluted to 1 M urea with 200 mM ammonium bicarbonate for trypsin digestion (1 µg, 37 °C, 8 h, Promega cat # V5113). After digestion, the peptide mix was acidified with formic acid and desalted with a MicroSpin C18 column (The Nest Group, Inc) prior to LC–MS/MS analysis.

### Chromatographic and Mass Spectrometric Analysis

Samples were analyzed using an LTQ-Orbitrap Fusion Lumos mass spectrometer (Thermo Fisher Scientific, San Jose, CA, USA) coupled to an EASY-nLC 1000 (Thermo Fisher Scientific (Proxeon), Odense, Denmark). Peptides were loaded directly onto the analytical column and were separated by reversed-phase chromatography using a 50-cm column with an inner diameter of 75 μm, packed with 2 μm C18 particle spectrometer (Thermo Scientific, San Jose, CA, USA). Chromatographic gradients started at 95% buffer A and 5% buffer B with a flow rate of 300 nl/min for 5 min and gradually increased to 25% buffer B and 75% A in 52 min and then to 40% buffer B and 60% A in 8 min. After each analysis, the column was washed for 10 min with 10% buffer A and 90% buffer B. Buffer A: 0.1% formic acid in water. Buffer B: 0.1% formic acid in 80% acetonitrile. The mass spectrometer was operated in positive ionization mode with nanospray voltage set at 2.4 kV and source temperature at 275 °C. Ultramark 1621 was used for external calibration of the FT mass analyzer prior to the analyses, and an internal calibration was performed using the background polysiloxane ion signal at m/z 445.1200. The acquisition was performed in data-dependent acquisition (DDA) mode, and full MS scans with 1 micro scan at a resolution of 120,000 were used over a mass range of m/z 350–1500 with detection in the Orbitrap mass analyzer. Auto gain control (AGC) was set to 1E5, and charge state filtering disqualifying singly charged peptides was activated. In each cycle of data-dependent acquisition analysis, following each survey scan, the most intense ions above a threshold ion count of 10,000 were selected for fragmentation. The number of selected precursor ions for fragmentation was determined by the “Top Speed” acquisition algorithm and a dynamic exclusion of 60 s. Fragment ion spectra were produced via high-energy collision dissociation (HCD) at a normalized collision energy of 28%, and they were acquired in the ion trap mass analyzer. AGC was set to 1E4, and an isolation window of 1.6 m/z and a maximum injection time of 200 ms were used. Digested bovine serum albumin (New England Biolabs cat # P8108S) was analyzed between each sample to avoid sample carryover and to assure stability of the instrument, and QCloud [26] has been used to control instrument longitudinal performance.

### LC–MS/MS Data Analysis

Acquired spectra were analyzed using the Proteome Discoverer software suite (v1.4, Thermo Fisher Scientific) and the Mascot search engine (v2.6, Matrix Science) [27]. The data were searched against a Swiss-Prot human database (as in April 2019, 20,421 entries) plus a list [28] of common contaminants and all the corresponding decoy entries. For peptide identification, a precursor ion mass tolerance of 7 ppm was used for MS1 level, trypsin was chosen as enzyme, and up to three missed cleavages were allowed. The fragment ion mass tolerance was set to 0.5 Da for MS2 spectra. Phosphorylation of serine, threonine and tyrosine, oxidation of methionine, and N-terminal protein acetylation were used as variable modifications, whereas carbamidomethylation on cysteines was set as a fixed modification. Precursor areas of phosphorylated peptides were extracted with the Skyline-daily software (v20.1.1.83); median of the area for each condition and fold change was calculated. False discovery rate (FDR) in peptide identification was set to a maximum of 5%. SAINTexpress algorithm [29] was used to score protein–protein interactions.

### GST Pull-Down Assay

Recombinant GST-PHDs proteins (PHD1, PHD2, and PHD3) and GST-HIF were obtained according to standard protocols in BL21 bacterial cells and purified using Glutathione-Sepharose 4B (GE Healthcare).

### HIF-1α Hydroxylation Assay

HEK-293T cells were transfected with PHDs as indicated. After 24 h of transfection, cells were stimulated as indicated for 24 h. After that, PHDs were immunoprecipitated as described [30]. Recombinant human GST-HIF-1α protein (#ab48734, Abcam) and immunoprecipitated PHDs were incubated in the reaction buffer 50 mM Tris/HCl (pH 7.5), 1 mM DTT, 50 μM FeSO_4_, 5 mM ascorbate, and 200 µM oxoglutarate for 1 h at 30 °C, respectively. The prolyl hydroxylation reaction was stopped by adding Laemmli sample buffer and analyzed by immunoblot assays.

### Luciferase Assay

For EPO-Luc transactivation as a marker of HIF stabilization, NIH-3T3-EPO-luc cells, containing three copies of the HRE consensus sequence from the promoter of the erythropoietin gene fused to the luciferase gene, were seeded in 96-well plates and incubated with the PP2A inhibitors LB-100 (#S7537, Selleckchem, Houston, USA) or Okadaic Acid Sodium Salt (#459620 Merk) as indicated for 30 min before BAH compound was added. Luciferase activity was quantified using Dual-Luciferase Assay (#E1483, Promega, Madison, WI, USA) after 6 h of stimulation.

### qPCR Analysis

STHdh^Q7/Q7^ and STHdh^Q111/Q111^ cells were stimulated with BAH for 6 h; after that, mRNA was extracted with High Pure RNA Isolation Kit (Roche, Barcelona, Spain). For *in vivo* experiments, total RNA was isolated from mice brain tissue using QIAzol lysis reagent and the RNeasy Lipid mini kit (#74,804, Qiagen, Hilden, Germany). For quantitative reverse transcriptase-PCR assays, total RNA (1 µg) was retrotranscribed using the iScript cDNA Synthesis Kit (#1,708,891, Bio-Rad, Hercules, CA, USA) and the cDNA was analyzed by real-time PCR using the iQTM SYBR Green Supermix (#1708880 Bio-Rad) and a CFX96 Real-time PCR Detection System (Bio-Rad). *Gapdh* gene was used to standardize mRNA expression in each sample. Gene expression was quantified using the 2^-ΔΔCt^ method, and the percentage of relative expression against controls (untreated cells or mice) was represented. Primer sequences are available upon request.

### Striatal Neuroprotection *in vitro*

STHdh^Q7/Q7^ and STHdh^Q111/Q111^ cells (10^4^ cells/well) were seeded in DMEM supplemented with 10% FBS in 96-well plates incubated with increased concentrations of BAH, and 30 min later, 3-NP at 10 mM (#N5636, Merk) was added. Then, 3-NP-induced cytotoxicity was measured by fluorescence using the die YOYO-1 (#Y3601, Life Technologies). Treated cells were placed in an Incucyte FLR imaging system, and the YOYO-1 fluorescence was measured after several time points. Object counting analysis is performed using the Incucyte FLR software to calculate the total number of YOYO-1 fluorescence positive cells and total DNA containing objects (endpoint). The cytotoxicity index is calculated by dividing the number of YOYO-1 fluorescence positive objects by the total number of DNA containing objects for each treatment group.

### Mice Model of Striatal Neurodegeneration

All animal experiments were performed in accordance with the European Union guideline and approved by the Animal Research Ethics Committee of Cordoba University (2014PI/017). Systemic administration of 3-nitropropionic acid (3-NP), an inhibitor of the mitochondrial complex II, results in a progressive locomotor deterioration and striatal degeneration resembling HD in different mice strains [31]. Striatal neurodegeneration was induced by seven intraperitoneal injections (i.p.) of 3-NP (50 mg/kg) every 12 h in sixteen-week-old C57BL/6 male (Envigo, Barcelona, Spain). Control mice received seven i.p. PBS injections. The treatment was administered daily by i.p. of BAH (30 mg/kg) or vehicle (1:1:18 ethanol: cremophor: saline). Twelve hours after the last administration of 3-NP, behavioral analyses were carried out by measuring hind limb clasping, hind limb dystonia, truncal dystonia, and general locomotor activity as previously described [32]. Each mouse was given a score of 0, 1, or 2 for each test, where 0 corresponds to normal behavior and 2 with the maximum motor disorder. Animals were sacrificed by cervical dislocation and brains were dissected, one hemisphere was immediately frozen and kept at −80 °C for RT-PCR analysis, and the other hemisphere was fixed in 4% formaldehyde.

### Histological Analysis

Immunohistochemical staining was performed on formalin-fixed, paraffin-embedded samples. Sections of 5 μm were rehydrated in a graded ethanol series. Nissl staining was performed using Cresyl-violet (#C5062, Merk); four random fields of each brain section were photographed, digitalized using a LeicaDFC420c camera, and analyzed using Image J software in a blinded manner by two independent observers.

### Immunohistochemistry Analysis

For IHC analysis, brain Sects. (5 µm) were deparaffinized and boiled for 10 min in sodium citrate buffer (10 mM, pH 6.0). Endogenous peroxidase activity was inhibited with 3.3% hydrogen peroxide in methanol. The sections were blocked with blocking reagent (#20773, Merck) and then incubated overnight at 4 °C with the following primary antibodies: rabbit anti-Iba-1 (1:100, #019–19741 Wako) and mouse anti-NeuN (1:100, #MAB377, Millipore). For blocking endogenous mouse IgG and non-specific background, rodent block M (#RBM961, Biocare Medical, Concord, CA) was used prior anti-NeuN antibody. Slides from anti-Iba-1 and anti NeuN were incubated with Vectastain Elite ABC HRP kit (#416411, Vector Laboratories) followed by secondary antibody (#21538-M, Merk; #BP-9100–50, Vector Laboratories) and were finally visualized with diaminobenzidine chromogen (Dako, Santa Clara, CA), photographed and digitalized using a Leica DFC420c camera, and analyzed using Image J software (http://rsbweb.nih.gov/ij/).

### Confocal Analysis

For antigen retrieval, paraffin-embedded brain Sects. (5-μm-thick) were deparaffinized and boiled for 10 min in sodium citrate buffer (10 mM, pH 6.0). The sections were washed three times in PBS-triton X100-saponin (0.1%). Nonspecific antibody-binding sites were blocked for 1 h at room temperature with 3% bovine serum albumin (BSA). Next, the sections were incubated overnight at 4 °C in the following primary antibody diluted in PBS with 3% BSA: mouse monoclonal anti-GFAP (1:100 dilution, #33673, Santa Cruz, Dallas, TX, USA). After extensive washing in PBS, slides were incubated with secondary antibodies for 1 h at room temperature in the dark. The immunoreactions were revealed using anti-mouse Alexa 488 (1:500 dilution, #A11029, Thermo Fischer Scientific, Waltham, MA, USA). Tissue sections were then mounted with Vectashield Antifade Mounting Medium with DAPI (H-1200, Vector Laboratories, Burlingame, Ca, USA). All images were acquired using a spectral confocal laser-scanning microscope LSM710, (Zeiss, Jena, Germany) with a 25 × /0.8 Plan-Apochromat oil immersion lens and quantified in 10–15 randomly chosen fields using ImageJ software.

### Pharmacokinetics

This study was performed by Pharmacology Discovery Services Taiwan, Ltd. a company of the Eurofins group. The experiment was accepted in accordance with Eurofins validation Standard Operating Procedure. Briefly, male SD rats (180–250 g. BioLasco Taiwan under Charles River Laboratories Licensee) received a single bolus intravenous (IV) administration of BAH at 3 mg/kg formulated in 1:1:18 Ethanol: Cremophor® EL: Saline (0.9% NaCl (Saline; Sing-Tong, Taiwan), Cremophor® EL (Sigma, USA), Ethanol (Merck, Germany)). Plasma samples and whole brains from 3 animals per time point were collected at 0.5, 1, and 3 h after IV administration, and 3 animals for BAH-free plasma and brain were used. Plasma and brain samples were processed using acetonitrile precipitation and analyzed by LC–MS/MS. The quantitative analysis of plasma and brain samples was performed as previously reported [11]. After analysis, BAH plasma and brain concentrations versus time graph were generated and the brain/plasma ratio per time point was calculated.

### Statistical Analysis

All the *in vitro* data are expressed as the mean ± SD, and all the *in vivo* data are expressed as the mean ± SEM. One-way ANOVA followed by Tukey’s or Dunnett’s post hoc tests was used to determine the statistical significance. The level of significance was set at *p<*0.05. Statistical analyses were performed using GraphPad Prism version 6.00 (GraphPad, San Diego, CA, USA).

## Results

### BAH Modifies PHD Activity

**Fig. 1:**
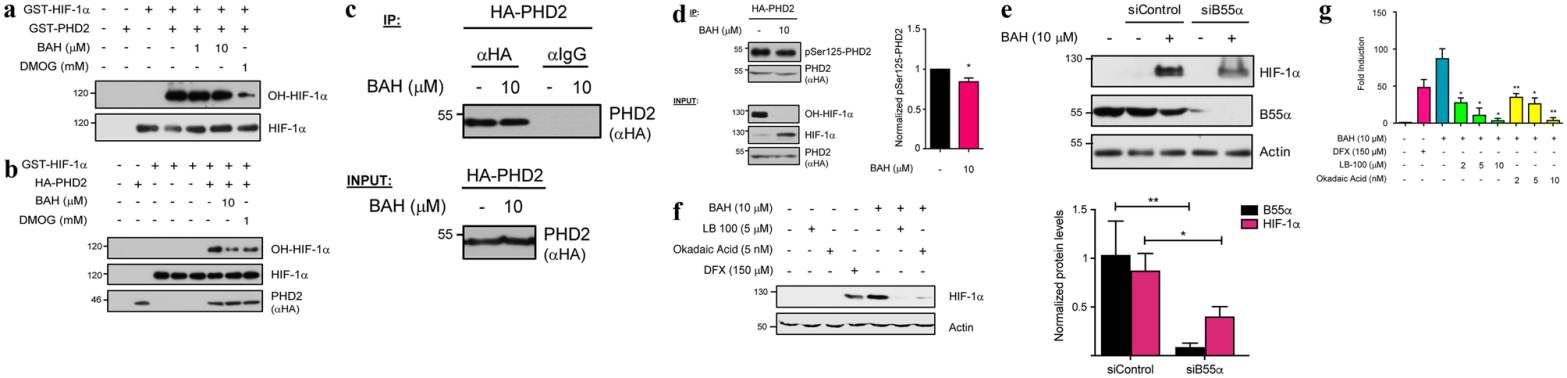
BAH alters PHD2 activity modulating Ser-125 Phosphorylation. **a** *In vitro* hydroxylation reactions were carried out employing recombinant GST proteins for HIF-1α and PHD2. Lanes 5th and 6th were treated with BAH. Hydroxylation levels of HIF-1α, as well as total HIF-1α signals, were analyzed by western blot. **b** HEK-293T cells were transfected with, HA-PHD2 plasmid. After 24 h, the indicated treatments with BAH or DMOG (PHDs inhibitor, positive control) were carried out. A total of 48 h after transfections, cell lysates were obtained and PHDs immunoprecipitated with specific anti-HA antibodies. Finally, these protein fractions were used to commit *in vitro* hydroxylation assays and hydroxylated HIF-1α levels were analyzed by immunoblotting. **c** HEK-293T cells were transfected with HA-PHD2 plasmid, treated with 10 μM BAH for 24 h and finally lysed. PHD2 protein was immunoprecipitated using either specific antibody (anti-HA) or an unspecific one (rat IgG), and its presence was observed by western blot. We present an Immunoprecipitation representation of the proteomic analyses performed (n = 3). **d** HEK-293T cells were transfected to express HA-PHD2, treated with BAH 50 μM for 24 h and lysed. A fraction was subjected to immunoprecipitation (IP) using an anti-HA antibody. After elution, phosphorylation was revealed with a specific anti-phospho Ser-125-PHD2 antibody, while exogenous HA-PHD2 protein levels were visualized with an anti-HA antibody by western blotting (top panel). The remaining extract fraction was tested for the occurrence of the indicated proteins (lower panel). Additionally, images were quantified (right) using ImageJ software (ImageJ 1.51 k, National Institutes of Health USA) and presented in a graph. We show a representative blot of three independent experiments. Data represent the mean ± SD (*n* = 3). *p < 0.05. **e** HEK-293T cells were transfected with B55α or scrambled (siControl) siRNAs, after 2 days in culture treated with BAH 10 μM for 6 h, and B55α and HIF-1α protein expression was analyzed by immunoblotting (representative blot of three independent experiments performed). Images were quantified (low) using ImageJ software, and HIF-1α and B55α protein levels were presented in a graph. Data represent the mean ± SD (*n* = 3). *p < 0.05. **f** HEK-293T cells were pretreated with PP2A inhibitors LB-100 (5 nM) and Okadaic Acid (5 nM) for 30 min. Then, treated with 10 μM BAH for 6 h and finally lysed. HIF-1α protein was detected using a specific antibody. We present a representative western blot of three independent analyses performed (*n* = 3). **g** NIH-3T3 fibroblasts stably transfected with a luciferase-HRE (NIH3T3-EPO-luc) were stimulated with the indicated concentrations of BAH, LB-100, and Okadaic Acid. Data represent the mean ± SD (n = 3). *p < 0.05, **p < 0.01

To identify the mechanism of action of BAH on the HIF pathway, we first analyzed its effect on PHDs activity *in vitro*. PHD recombinant protein hydroxylation activity on HIF-1α was determined in the presence or absence of BAH. As shown in Fig. 1A and Supplementary Fig. 1A–B, BAH did not affect the enzymatic activity of the PHDs *in vitro*. Similar results were obtained for PHD2 performed under more restrictive conditions in iron and ascorbic acid concentration (Supplementary Fig. 1C). Next, we decided to analyze the effect of BAH on the activity of immunoprecipitated PHDs of cells that had been previously stimulated with BAH. We found that treatment with BAH produced a clear inhibition in the capacity of PHD2 and PHD3 to hydroxylate HIF-1α (Fig. 1B; Supplementary Fig. 1E). However, no alteration in PHD1 hydroxylation activity was observed (Supplementary Fig. 1D). Altogether, these results suggest that BAH has the ability to modify PHD2 and PHD3 activity in an indirect way, without affecting either their binding with HIF-1α or through a direct interaction.

### BAH Affects Both Phosphorylation at Ser-125 and PHD2 Interactome

Next, we decided to analyze changes in interactome and PHD2 post-translational modifications (PTMs) after stimulation with BAH. To do this, PHD2 was immunoprecipitated from cells after stimulation with BAH (Fig. 1C), and PTMs and interactome were analyzed by LC–MS/MS. The identified peptides allowed a 78% coverture of PHD2 sequence. From the PTMs identified, a clear difference in the levels of Ser-125 phosphorylation was observed after treatment with BAH compared to a control situation (Supplementary Fig. 2). Then, and to confirm this observation, phosphorylation status was analyzed using a phospho-specific antibody. Results proved that stimulation with BAH produces a 14% reduction in Ser-125 phosphorylation (Fig. 1D). Previous studies have confirmed that the protein phosphatase 2A (PP2A/B55α) is considered one of the main modulators of Ser-125 PHD2 phosphorylation [13]. To verify whether BAH stimulation modifies Ser-125 PHD2 phosphorylation via PP2A/B55α dephosphorylation, we undertook a loss-of-function approach. B55α depletion by siRNA substantially decreased HIF-1α protein level after stimulation with BAH (Fig. 1E). In the same way, we employed the well characterized PP2A inhibitors LB-100 and Okadaic Acid (OA). As shown in Fig. 1F, HIF-1α protein level stimulation mediated by BAH clearly decreased after stimulation with LB-100 or OA. Additionally, this observation was further reinforced by measuring the downstream effects of HIF-1α (EPO induction) in the presence of both inhibitors. As shown in Fig. 1G, HIF-1α transcriptional activity mediated by BAH was dose-dependent compromised followed by increasing LB-100 and OA concentrations.

**Fig. 2:**
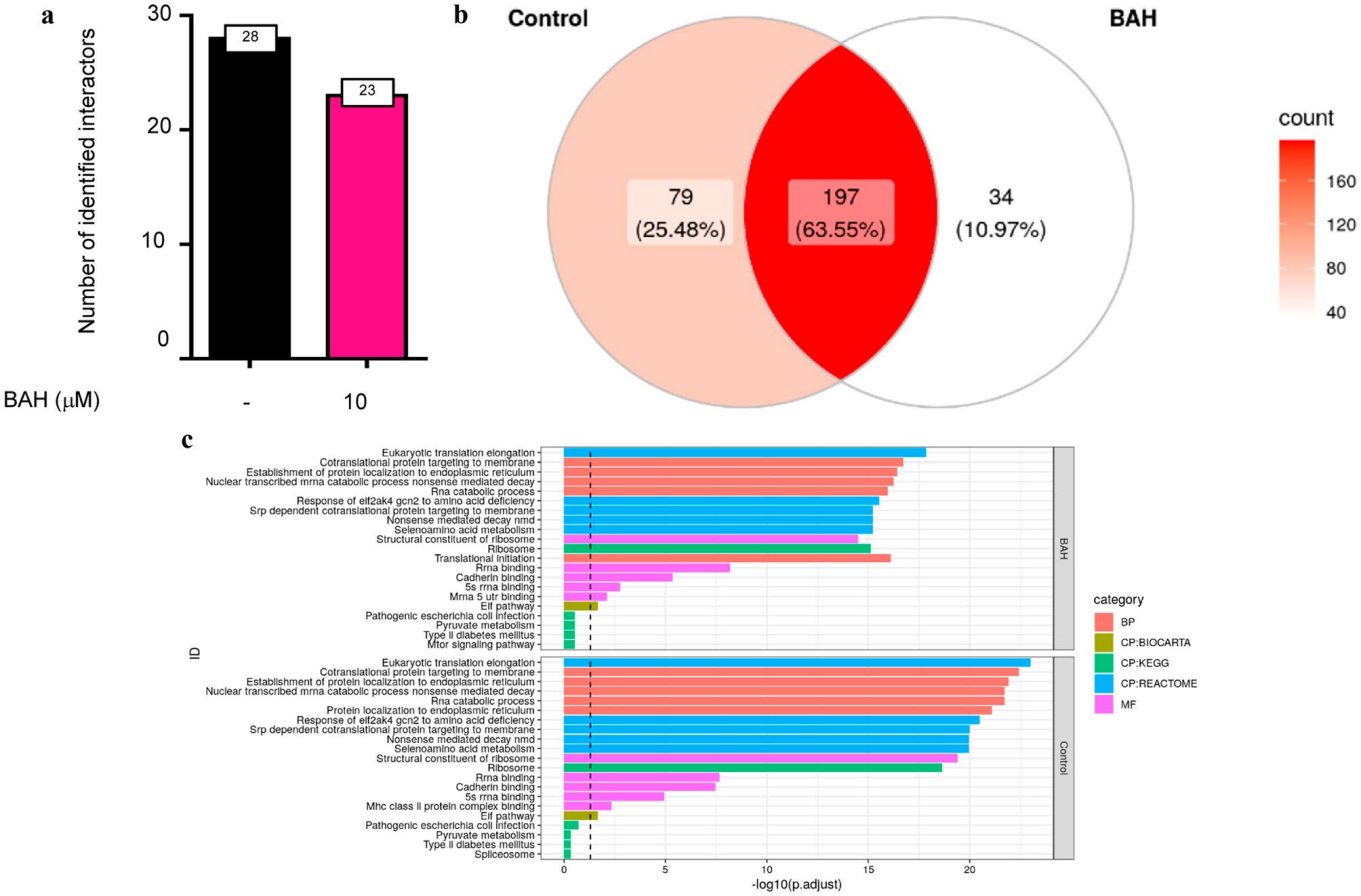
BAH affects PHD2 interactome. **a** Significative data of the interactome analyses performed: Graph bar shows the protein number of each group. **b** Venn diagram classifies the obtained proteins using MSigDb categories and individual interactors for control and BAH. **c** Plot TOP 5 over-represented pathways per category and treatment where labels indicate the number of overlapping genes in a given functional category and the length of the bar the significance of the enrichment. The dashed line indicates the consensus cutoff of *p* = 0.05

On the other hand, interactome results revealed that 277 identified proteins were interacting under normal conditions with PHD2, whereas 232 interactors were found after stimulation with BAH (Supplementary Table 1). To eliminate possible non-specific interactors, SAINT algorithm was employed with a 0.2 threshold to specifically select those proteins that are not present in an antibody immunoprecipitation control (IgG). After that restriction, the proteins obtained under control conditions were reduced to 28, whereas those found following treatment with BAH were 23 (Fig. 2A; Supplementary Table 2). Further treatment of the data enabled us to categorize these identified proteins into three different groups. The first group was characterized by those proteins present only in the untreated cells, which are consequently lost following treatment with BAH (28.12% of identified proteins). The second group was formed by those proteins that remained unaltered within the treatments (58.38%). Finally, the last group contained the proteins that appeared only after treatment with BAH (12.5%) (Fig. 2A). The subsequent functional characterization of these data through an over-representation analysis has allowed us to have a global vision of the contribution of the alteration of PHD2 interactome mediated by the stimulation with BAH on different cell functions, amongst which we can mention a clear reduction in pathways related to co-translational protein targeting to membrane, translation initiation or cell adhesion molecule binding (Fig. 2C).

### Effects of BAH on Striatal Neurons Expressing Normal and Mutated Huntingtin

**Fig. 3:**
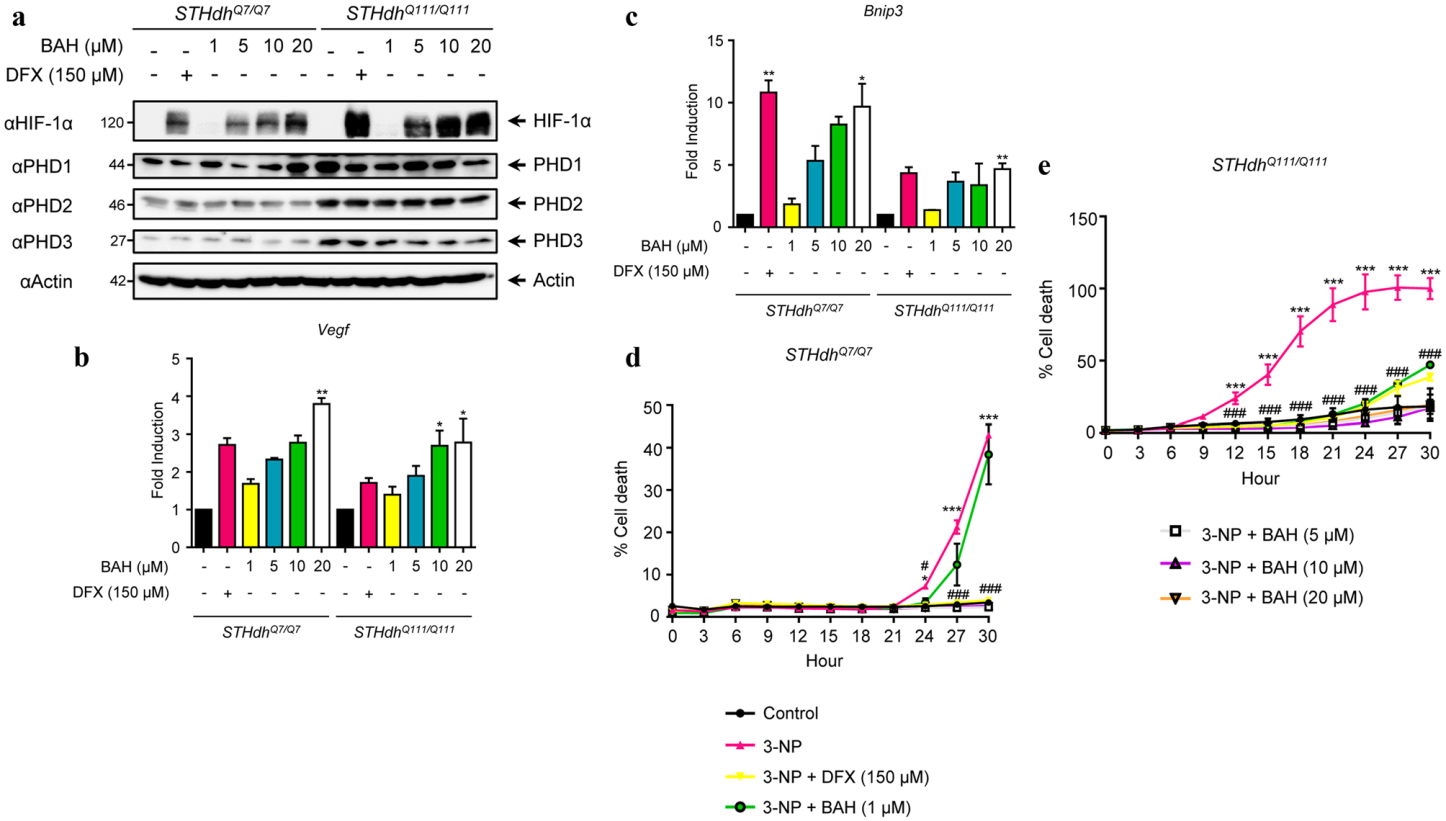
Effects of compound BAH on striatal cells harboring wild type and mutant huntingtin. **a** STHdh^Q7/Q7^ and STHdh^Q111/Q111^ cells were treated with BAH for 6 h and further analyzed for HIF-1α, PHD1, PHD2, and PHD3 expression by immunoblot. The mRNA expression levels of *Vegf-A* **b** and *Bnip3* **c** genes were quantified by qPCR in STHdh^Q7/Q7^ and STHdh^Q111/Q111^ cells after 6 h of treatment with the compound. Data represent the mean ± SD (*n* = 3). *p < 0.05, **p < 0.01, BAH-treated cells vs untreated cells (one-way ANOVA followed Dunnett’s test). **d** STHdh^Q7/Q7^ and STHdh^Q111/Q111^ cells were pre-incubated with the indicated concentrations of BAH for 6 h and stimulated with 3-NP for 30 h. The percentage of neuronal death was determined by YOYO-1 and referred to vehicle-treated neurons. Data represent the mean ± SD (*n* = 3). *p < 0.05, ***p < 0.001 3-NP treated cells vs untreated cells; #*p* < 0.05, ###*p* < 0.001 3-NP + BAH-treated cells vs 3-NP treated cells (two-way ANOVA followed Tukey’s test)

To study the effect of BAH on the HIF pathway, immortalized striatal neurons expressing normal huntingtin protein (STHdh^Q7/Q7^) or a mutant version associated with juvenile-onset of HD (STHdh^Q111/Q111^) [24] were stimulated with the indicated doses and protein expression of HIF-1α and PHDs were analyzed. As depicted in Fig. 3A, BAH stabilized HIF-1α protein in a concentration-dependent manner in neurons expressing normal and mutated huntingtin proteins. Although the basal expression of PHDs proteins is higher in STHdh^Q111/Q111^ cell line compared to STHdh^Q7/Q7^, no significant changes were found after treatment with BAH. To further investigate the activation of the HIF pathway, vascular endothelial growth factor (*Vegf*) and B cell leukemia/lymphoma 2 (Bcl-2)/adenovirus (E1B)-19KD-interacting protein 3 (*Bnip3*) gene expression was determined (Fig. 3B–C). In both cases, BAH was able to induce the expression of *Vegf* and *Bnip3* in a concentration-dependent manner. Finally, the neuroprotective effect of BAH was evaluated in response to mitochondrial dysfunction in neurons induced by the selective complex II inhibitor 3-nitropropionic acid (3-NP). As expected, 3-NP was strongly cytotoxic in STHdh^Q111/Q111^ cells compared to STHdh^Q7/Q7^ cells, and treatment with BAH protected both types of cells from 3-NP-induced cell death (Fig. 3D–E).

### Neuroprotective Effect of BAH in a Murine Model of Neuronal Striatal Degeneration

**Fig. 4:**
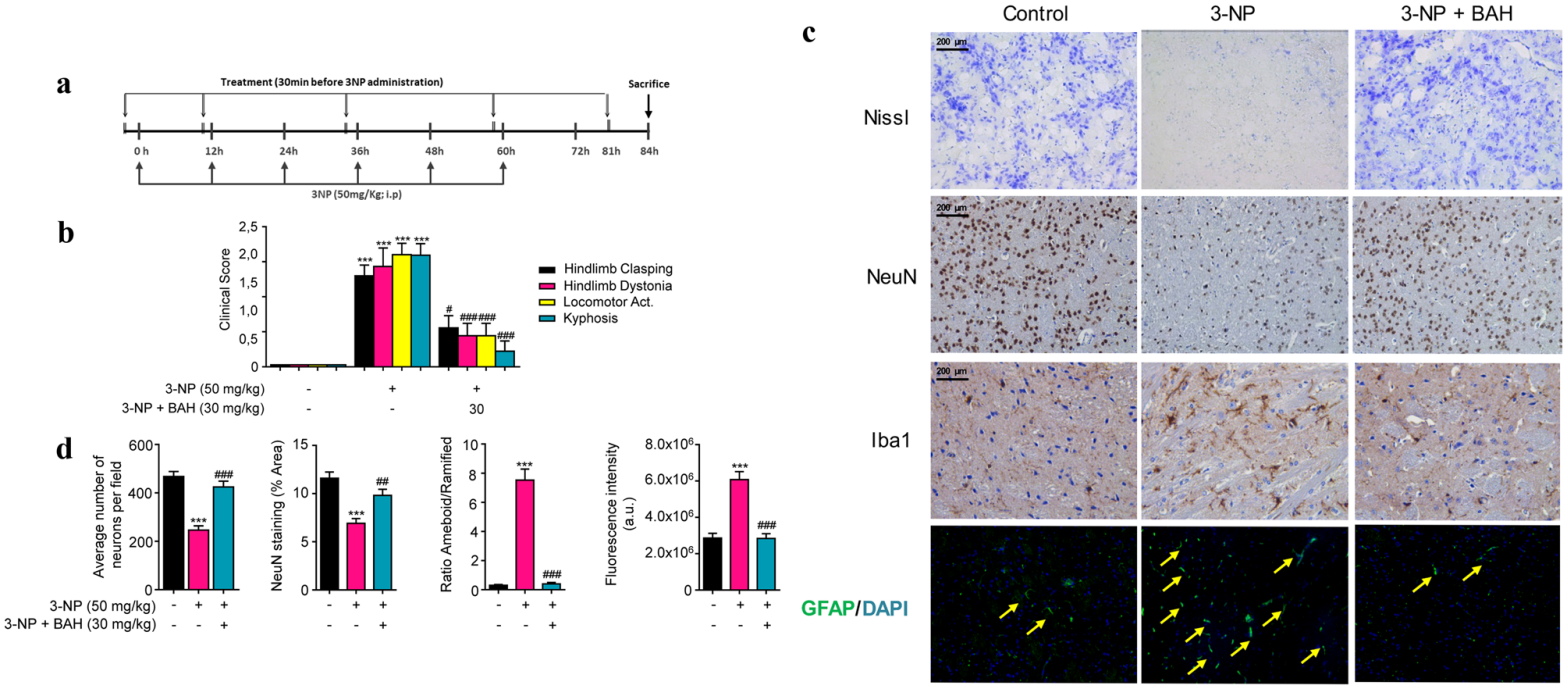
Compound BAH is neuroprotective in 3-NP-intoxicated mice. **a** Representative scheme of the experimental protocol used for the induction of HD in mice. **b** The behavioral score was determined 12 h after 3-NP intoxication. Mice were treated with BAH (30 mg/kg) as indicated. Hind limb clasping, general locomotor activity, hind limb dystonia, and kyphosis were rated from 0 to 2 based on severity, with score 0 indicating normal function, and 2 indicating the most severe affected function. Values are expressed as means ± SEM (*n* = 6). ***p < 0.001 3-NP vs. Vehicle; #*p* < 0.05, ###*p* < 0.001 3-NP + BAH vs 3-NP (one-way ANOVA followed Tukey’s test). **c** Nissl staining, NeuN, and Iba-1 immunohistochemistry and immunofluorescence labeling of GFAP (green fluorescence) in coronal striatal brain sections from the different mice groups. **d** Quantifications of Nissl, NeuN, Iba1, and GFAP expression (yellow arrows) were performed with Image J software. Values are expressed as mean ± SEM (*n* = 6). ***p < 0.001 3-NP vs. Vehicle; ###*p* < 0.001 3-NP + BAH vs 3-NP (one-way ANOVA followed Tukey’s test)

In order to confirm the neuroprotective action of BAH *in vivo*, a model of Huntington’s disease based on 3-NP administration was used (Fig. 4A). Treatment with 3-NP results in several alterations, including neurological and histological changes characteristic of some aspects of HD pathology. Compared to control animals treated with vehicle, the 3-NP-treated mice exhibited high scores in hind limb clasping, locomotor activity, hind limb dystonia, and kyphosis (Fig. 4B) which were improved after the treatment with BAH. We next investigated the impact of BAH in striatal degeneration and atrophy. The administration of 3-NP reduced the number of neurons in the striatum, as determined by Nissl staining and NeuN immunohistochemistry (Fig. 4C–D). In addition, BAH-mediated neuroprotection was associated with reduced 3-NP-induced astrogliosis as determined by GFAP immunofluorescence and a decrease in the activated microglia. 3-NP mice showed the associated change of morphology typical of microglial activation with the cytoplasmatic ramifications retracted and an ameboid appearance [33] whereas Iba1-immunopositive cells from control mice and those treated with BAH exhibited a ramified morphology with extensive branching and processes.

**Fig. 5:**
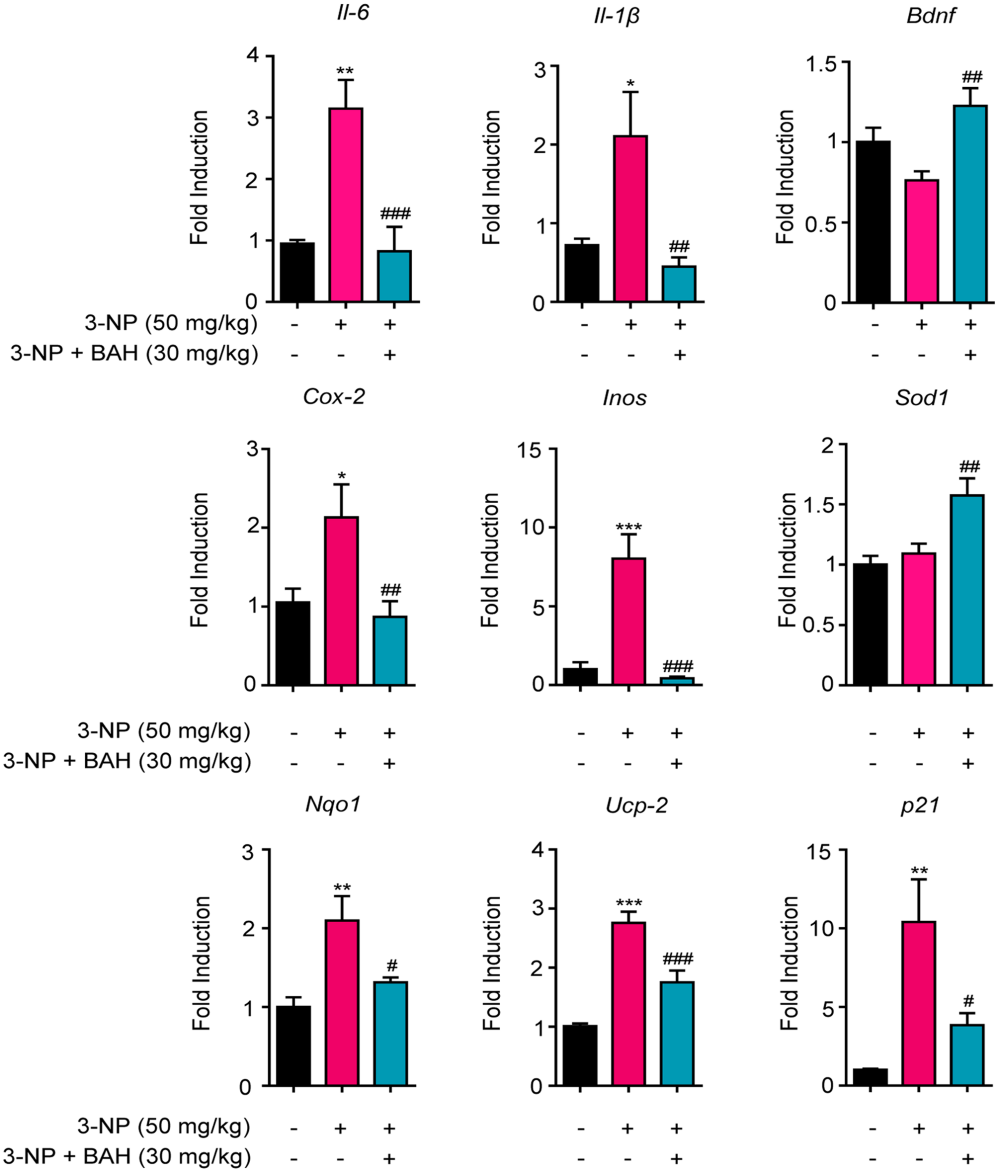
Treatment with BAH induces changes in gene expression. Gene expression of inflammatory markers including *Il-6*, *iNos*, *Cox-2*, and *Il-1β* was significantly downregulated in 3-NP + BAH-treated mice compared with 3-NP mice, whereas *Bdnf* and *Sod1* expression was the opposite. Neuroprotection markers such as *Nqo1*, *Ucp-2*, and *p21* also altered their expression after 3-NP insult. Values are expressed as means ± SEM (*n* = 6 animals per group). **p < 0.01, ***p < 0.001 3-NP vs. Vehicle; #*p* < 0.05, ##*p* < 0.01, ###*p* < 0.001 3-NP + BAH vs 3-NP (one-way ANOVA followed Tukey’s test)

We also analyzed the expression of specific pro-inflammatory markers. In 3-NP-lesioned mice, upregulation of mRNA levels of inflammatory markers such as *Cox-2*, *iNos*, *Il-1*, and *Il-6* was observed and fully inhibited by treatment with BAH (Fig. 5). In addition, other genes related to oxidative stress and neuroprotection were studied. *Bdnf* and *Sod1* genes which are associated with neuroprotection [34] were enhanced in mice treated with BAH, whereas others such as *Nqo1*, *Ucp-2*, and *p21* had their expression increased in 3-NP-lesioned mice but almost recovered their basal levels after the treatment with the compound BAH.

**Fig. 6:**
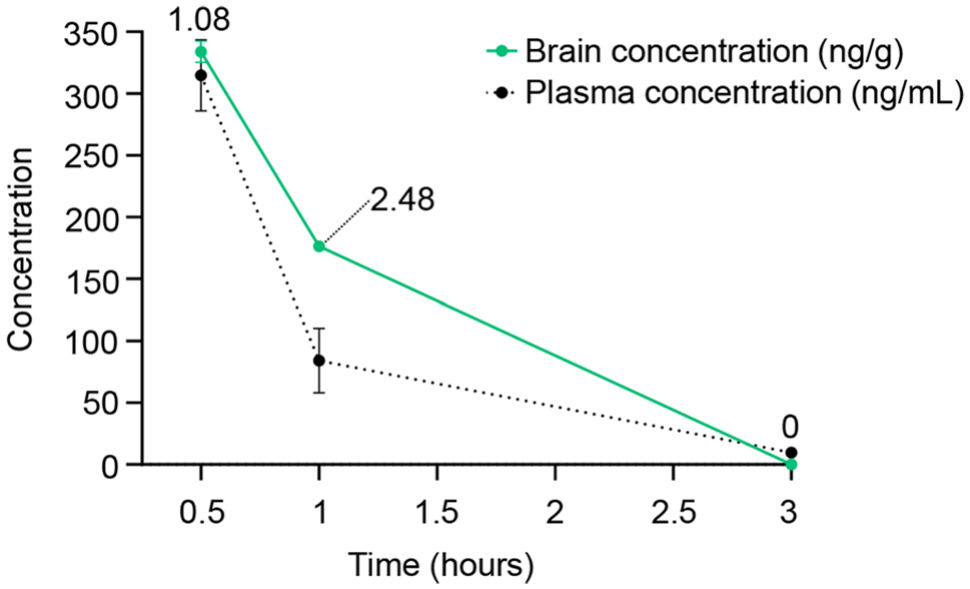
BAH brain penetration. Plasma and brain pharmacokinetic profiles of BAH in 1:1:18 Ethanol: Cremophor® EL: Saline given by i.v. route to male SD rats (3 mg/kg; *n* = 3 rat per time, mean ± SEM) (brain concentration—green dot—in ng/g and plasma concentration—black dot—in ng/mL). Values of brain/plasma ratios per time point are indicated in the graph

As BAH exerted a neuroprotective action *in vivo*, we evaluated its brain penetration. Pharmacokinetic analyses were performed in rats after administration of BAH (2 mg/kg). The highest plasmatic levels for BAH were detected after 30 min and then rapidly declined at 120 min indicating a rapid excretion. Importantly, the levels of BAH in the brain were similar to those in the plasma, indicating a very good brain penetration and not accumulative effect was found at the time point studied (Fig. 6).

## Discussion

Neurodegenerative diseases such as HD are characterized by a progressive deterioration in the structure and function of the brain. Its incidence has raised significantly mainly because of the increase in life expectancy, representing a major health problem that demands new therapeutic strategies for their treatment. Accumulating data indicate that mild HIF-1α activation (hypoxia preconditioning) protects the brain [35] against several types of injury such as ROS [36] and inflammation [37] through the induction of adaptive mechanisms that involve the modulation of glucose transporters, glycolytic enzymes, and angiogenic factors. Moreover, HIF-1α-mediated upregulation of *Bnip3* has been shown to mediate neuroprotection by activating the autophagy pathway [38].

Our results suggest that BAH has the ability to modify PHD2 activity without affecting either their binding with HIF-1α or through a direct interaction. The PHD2 Ser-125 phosphorylation clear reduction obtained in MS/MS, added to its validation using a phospho-specific Ser-125-PHD2 antibody, indicates that BAH reduces this residue phosphorylation. In this sense, it has been previously described that Ser-125 phosphorylation increases PHD2 activity leading to a higher HIF-1α degradation. Moreover, this phosphorylation status is accurately controlled by the P70S6K kinase and PP2A phosphatase [13]. Although further studies should be done to clarify how BAH modifies PHD2 phosphorylation status, we found that pan-PP2A inhibitors LB-100 and OA inhibited BAH-induced HIF-1α stabilization. PP2A-activating drugs (PADs) are being actively sought and investigated not only as potential anti-cancer treatments but also against inflammatory and neurodegenerative diseases [39]. PP2A is a highly complex heterotrimeric enzyme that catalyzes the selective removal of phosphate groups from protein serine and threonine residues and further experiments are warranted to identify the PP2A subtype involved in the mechanism of action for BAH.

HIF-1α also upregulates a plethora of genes including vascular endothelial growth factor (*VEGF*) and erythropoietin, both showing neuroprotective activity in different animal models of neurological diseases. Although high levels of *VEGF* are found significantly increased in HD patients when compared with the controls [40], and there is evidence that *VEGF* causes BBB disruption [40, 41], other reports indicate that *VEGF* itself has neurotrophic effects, as it stimulates axonal outgrowth and increases the survival of neurons at different regions of the brain [42], and also rescues hippocampal cells from death induced by serum withdrawal [43]. In addition, HIF PHD inhibition also protects cortical neurons from 3-NP-induced cytotoxicity that correlates with enhanced *VEGF* expression [20]. Furthermore, low doses of *VEGF165* are neuroprotective in *in vitro* and *in vivo* models of HD caused by overexpression of mutated huntingtin [44].

Betulinic acid (BA) is a pleiotropic bioactive compound that has been described to act on different molecular targets that mediated neuroprotection and antiinflammatory activity. It is likely that distinct pharmacophores in the BA molecule are responsible for the activity on different targets and therefore BAH would retain some of the activities attributed to BA. Thus, one of the possible mechanisms from which BAH could carry out its actions would be decreasing the inflammatory response. A number of studies indicate that activation of the immune system and an altered immune response are evident even in the premanifest stage of Huntington’s disease [45]. In fact, activated microglia and reactive astrocytes contribute to neural death in HD pathology [46]. Immunohistochemical analysis of the striatum from mice treated with 3-NP showed that BAH treatment reduced the expression of GFAP, the main biomarker of reactive astrogliosis [47]. In addition, activated microglia, which is clearly present in intoxicated 3-NP mice, turned into a resting state when treated with BAH compound. These results are in accordance with the decrease of the expression of proinflammatory cytokines (*Il-1β*, *Il-6*, *iNos*, and *Cox-2*) observed by RT-PCR, suggesting that BAH would be able to reduce the cascade of proinflammatory cytokines induced by activated microglia and astrogliosis [48]. In addition, we also investigated the expression levels of the brain-derived neurotrophic factor (*Bdnf*), a critical survival factor for striatal neurons that die in HD [49]. Results showed a slight decrease in its level after 3-NP administration, as previously described [50], but a significant increase in mice treated with BAH compound, suggesting other mechanism by which BAH could induce neuroprotection.

Finally, oxidative stress and mitochondrial dysfunction have been linked to neurodegenerative disorders including HD [51, 52]. Previous reports indicated that HIF PHDs inhibitors protected striatal cells bearing a mutated form of the huntingtin protein against mitochondrial toxin-induced cytotoxicity [20]. Our results corroborate these publications, showing a remarkable protection from 3-NP in STHdh^Q111/Q111^ cells treated with BAH since the lowest concentration (1 μM). In the mice model, HIF-1α-dependent genes involved in responses to stress with neuroprotective actions were studied. *Nqo1*, *p21*, and *Ucp-2* gene expressions were increased in those mice treated with 3-NP, indicating a stress situation. However, a decrease in the level of these genes after BAH treatment prompted us to believe that our compound produced neuroprotective actions.

In summary, the hypoximimetic triterpenoid derivative BAH was able to alter the response pathway to hypoxia through the decrease of PHD2 phosphorylation levels. In addition, the compound provided neuroprotection in a striatal degeneration mice model induced by 3-NP, improving the clinical symptoms and antioxidant defenses in the brain, preventing neuronal loss, decreasing reactive astrogliosis and microglial activation, and decreasing the expression of proinflammatory markers. These results indicated that BAH has the potential for further pharmacological development of a novel drug candidate for the treatment of HD and perhaps other neurodegenerative diseases.

## Supplementary Information

Below is the link to the electronic supplementary material.

Supplementary file1 (PDF 206 KB)

Supplementary file2 (PDF 5665 KB)

Supplementary file3 (PDF 470 KB)

Supplementary file4 (PDF 508 KB)

Supplementary file5 (PDF 471 KB)

Supplementary file6 (PDF 467 KB)

Supplementary file7 (PDF 473 KB)

Supplementary file8 (PDF 463 KB)

Supplementary file9 (PDF 467 KB)

Supplementary file10 (DOCX 1337 KB)

Supplementary file11 (PDF 469 KB)

Supplementary file12 (XLSX 469 KB)

Supplementary file13 (XLSX 469 KB)

## Data Availability

The raw data supporting the conclusions of this article will be made available by the authors, without undue reservation.
