# Supplementary material for: Betulinic Acid Hydroxamate is Neuroprotective and Induces Protein Phosphatase 2A-Dependent HIF-1α Stabilization and Post-transcriptional Dephosphorylation of Prolyl Hydrolase 2
Source: Neurotherapeutics. 2021 Aug 2;18(3):1849–61. doi: 10.1007/s13311-021-01089-4 (PMC8608974; doi:10.1007/s13311-021-01089-4)
Supplement: Supplementary file 10 — Supplementary file10 (DOCX 1337 KB) [file 13311_2021_1089_MOESM10_ESM.docx]

**Supplementary figures.**

**Supplementary Figure S1.**

**(A-B).** *In vitro* hydroxylation reactions were carried out employing recombinant GST proteins for HIF1α and PHDs. Lanes 5th and 6th were treated with BAH and lane 7th was treated with BA as a positive control. Hydroxylation levels of HIF1α as well as total HIF1α signals were analyzed by western blot. (**C**). *In vitro* hydroxylation reactions were carried out as in Figure 1A but with lower concentration of FeSO_4_ (10 μM) and ascorbate (100 μM). **(D-E).** HEK-293T cell were transfected with either HA-PHD1 or HA-PHD3 plasmids. After 24 h the indicated treatments with BAH, BA or DMOG (PHDs inhibitor, positive control) were carried out. 48 h after transfections, cell lysates were obtained and PHDs immunoprecipitated with specific anti-HA antibodies. Finally, these protein fractions were used to commit *in vitro* hydroxylation assays and hydroxylated HIF1α levels were analyzed by immunoblotting.

**Supplementary Figure S2.**

**A**. Schematic representation of mass spectrometry (MS) analysis results. Coverage of the protein sequence was 78.17 %. A red box within the PHD2 sequence (101-150) points out phosphorylation found at Ser125 in control experiment, which reduces its signal under BAH treatment. **B.** Summary graphic that shows the spectrum Ser125 phosphopeptide retention time from the immunoprecipitation assay coupled to mass spectrometry. **C.** Ser125 phosphopeptide peak area of the three replicates of the mass spectrometry assay.

+

+

+

+

+

+

+

-

2

-

-

-

5

10

-

-

-

-

2

5

10

BAH (10 µM)

-

-

-

-

-

-

-

LB-100 (μM)

-

2

-

-

-

5

10

Okadaic Acid (nM)

-

-

-

-

2

5

10

**Supplementary Figure S3.**

HEK-293T cells were seeded at a density of 1,2 x 10 ^4^ cells/well in 96-well plates. After 24 hours, cells were treated with increasing concentrations of PP2A inhibitors (LB-100 or Okadaic Acid) in presence or absence of 10 mμM BAH for 2 hours. Then, 50 μl of 3-MTT 5 mg/ml (4,5-Dimethylthiazol-2-yl)-2,5-diphenyltetrazolium bromide) (Sigma-Aldrich) from a mixture solution of MTT: DMEM (1:2) per well was added and cells were incubated for 4 hours at 37 °C in darkness. Finally, supernatant was removed and 100 μl DMSO was incorporated to each well for 10 min, in gentle shaking. Absorbance was measured at 550nm using a TriStar LB 941 (Berthold Technologies, GmbH & Co. KG). The mean concentration in each set of three wells was measured. The absorbance of untreated controls was considered as 100% survival. Here, we present a graphical representation of n=3 biological different experiments.
